# The Genetics of Deafness in Domestic Animals

**DOI:** 10.3389/fvets.2015.00029

**Published:** 2015-09-08

**Authors:** George M. Strain

**Affiliations:** ^1^Comparative Biomedical Sciences, School of Veterinary Medicine, Louisiana State University, Baton Rouge, LA, USA

**Keywords:** piebald, merle, pigmentation, cochlea, KIT, MITF, PMEL, EDNRB

## Abstract

Although deafness can be acquired throughout an animal’s life from a variety of causes, hereditary deafness, especially congenital hereditary deafness, is a significant problem in several species. Extensive reviews exist of the genetics of deafness in humans and mice, but not for deafness in domestic animals. Hereditary deafness in many species and breeds is associated with loci for white pigmentation, where the cochlear pathology is cochleo-saccular. In other cases, there is no pigmentation association and the cochlear pathology is neuroepithelial. Late onset hereditary deafness has recently been identified in dogs and may be present but not yet recognized in other species. Few genes responsible for deafness have been identified in animals, but progress has been made for identifying genes responsible for the associated pigmentation phenotypes. Across species, the genes identified with deafness or white pigmentation patterns include *MITF*, *PMEL*, *KIT*, *EDNRB*, *CDH23*, *TYR*, and *TRPM1* in dog, cat, horse, cow, pig, sheep, ferret, mink, camelid, and rabbit. Multiple causative genes are present in some species. Significant work remains in many cases to identify specific chromosomal deafness genes so that DNA testing can be used to identify carriers of the mutated genes and thereby reduce deafness prevalence.

Auditory function plays a significant role in domesticated animals as they interact with conspecifics, other animals, and humans, and in non-domesticated animals as they interact with conspecifics, prey species, and predator species. Diminished or absent audition can present danger or even be lethal, whether it be a deaf dog struck by an undetected motor vehicle, or a deaf wild animal succumbing to an undetected predator.

Deafness or hearing loss can be classified by a variety of criteria (1). Hearing loss can be unilateral or bilateral, depending on affected ears (2). Hearing loss can be partial or total, based on the extent of loss (3). It can be syndromic or non-syndromic, based on whether other system diseases or phenotype abnormalities accompany the loss. Finally, (4) hearing loss or deafness can be peripheral, involving the outer, middle or inner ear, or central, where the pathology is retrocochlear. This review will focus on peripheral deafness, where many of the other classification criteria listed above provide further clarifying descriptions.

Peripheral deafness in turn can be classified based on a number of criteria: (1) it can be inherited or acquired; (2) it can be congenital or late onset; or (3) it can be conductive, where hearing loss results from decreased sound transmission through the outer and middle ear, or sensorineural. These three pairs of descriptors combine into specific forms of peripheral deafness, such as hereditary/congenital/sensorineural deafness, or acquired/late onset/sensorineural deafness, or acquired/congenital/conductive deafness. Congenital deafness is usually hereditary, but may result from other causes. The classifications and examples of forms of peripheral deafness are listed in Table 1. Numerous publications have reviewed the genetics of deafness in humans and mice (1–15) and deafness in dogs and cats (16, 17). This review will focus on known or suspected hereditary forms of peripheral deafness in domestic species. Studies of rodent species utilized in auditory research applications are beyond the scope of this review and so will only be touched on where relevant. For consistency, traditional Mendelian genes based on phenotype will be referred to as loci, while chromosomal locations identified by molecular genetic approaches will be referred to as genes.

**Table 1:** **Classification and examples of different types of peripheral deafness**.

	Sensorineural		Conductive	
	Congenital	Late onset	Congenital	Late onset
Hereditary	Pigment-associated (CS)^a^ – Dalmatian Non-pigment-associated (NE)^a^ – Doberman pinscher, Puli	Border collie, Rhodesian ridgeback	None known	Primary secretory otitis media^b^ – Cavalier King Charles spaniel
Acquired	Perinatal anoxia Dystocia Intrauterine ototoxin exposure – gentamicin	Ototoxin exposure – gentamicin Otitis interna Presbycusis Noise trauma Physical trauma Anesthesia-associated Undetermined	Ear canal atresia	Otitis externa Otitis media Cerumen impaction Ear canal inflammation Ear canal foreign bodies – awns Middle ear polyps

*^a^CS, cochleo-saccular; NE, neuroepithelial*.

*^b^Hereditary basis suspected but not confirmed*.

## Pathology

An understanding of the forms of hereditary deafness greatly benefits from a logical classification system based on the type of pathology present. An early attempt by Ormerod (18) was based on human histological specimens, but the categories were mostly based on limited numbers and proved unsatisfactory. Grűneberg (19) in an earlier study had categorized deaf mouse mutants into (1) those with morphogenetic abnormalities that reflected developmental abnormalities and (2) those with degenerative abnormalities of the sensory epithelia of the inner ear occurring after normal development but during the postnatal period. Steel (20–22) developed a classification system following both earlier studies with three types of pathology that cover observed forms of deafness in a more satisfactory manner. These include a morphogenetic pathology similar to that of Grűneberg, and two types of degenerative pathologies, including one (Scheibe type) described by Ormerod.

### Morphogenetic

This category includes all cases of structural abnormalities of the membranous and bony labyrinths, up to and including a complete absence of auditory and/or vestibular components. The effects of this pathology on hearing range from profound deafness to no hearing loss, which nevertheless is accompanied by effects on vestibular structures.

### Neuroepithelial

Neuroepithelial (NE) pathology exhibits loss of cochlear hair cells as the primary event. Degeneration begins when normal histological development of the organ of Corti is finishing. Outer hair cells degenerate before inner hair cells, beginning in the upper part of the basal turn and progressing to both apex and base (21). Supporting cells of the organ of Corti collapse, leaving only the basilar membrane and dedifferentiated cells. The stria vascularis and Reissner’s membrane are initially unaffected and all vestibular structures can be affected in addition to the cochlea. The expression of this pathology is typically bilateral. An endocochlear potential may still be present in the cochlear duct. This pathology has been reported in mice (18), guinea pigs (23, 24), dogs (25–27), and humans (28).

### Cochleo-saccular

Also known as Scheibe type pathology, this condition results from a primary degeneration of the stria vascularis on the outer wall of the cochlear duct, proceeding to degeneration of hair cells, collapse of Reissner’s membrane and occasional collapse of the saccule, with collapse of the saccule wall and macula damage, without effect on the remainder of the vestibular structures. The expression is more often unilateral but may affect both ears. No endocochlear potential remains. The initial strial degeneration is usually linked to the absence of functioning melanocytes, and as a result may be described as pigment-associated. CS pathology has been reported in the white cat (29, 30), Dalmatian (31–33) and other dog breeds (34, 35), Hedlund white mink (36, 37), several mouse mutants (38, 39), and as Waardenburg syndrome in humans (40). As background, the normal postnatal maturation of the stria vascularis has been described for the dog (41) and the functional morphology of melanocyte types has been described for the mouse (42).

## Deafness Statistics in Humans

Impairment of hearing is the most common sensory deficit in humans, affecting more than 250 million people in the world (43); however, similar data are not available for animal species. Congenital deafness or hearing loss occurs in one to six of every 1,000 human births, with at least one case in every 2,000 births being genetic (12). Roughly 20% of congenital deafness is non-hereditary, often due to a condition known as Mondini dysplasia (44). Among humans, 50–60% of all deafness in children is genetic (45); hereditary late onset hearing loss also occurs. Hereditary deafness may be non-syndromic autosomal recessive (AR) (8), non-syndromic autosomal dominant (AD) (6), sex-linked (7), mitochondrial (4, 9), or syndromic; it may also be monogenic or polygenic (20, 46), although little is known about the latter. About 80% of genetic hearing loss is non-syndromic (12), with 60–75% of those being AR, 20–30% AD, and 2% X-linked or mitochondrial (1). Note that the percentages above may not add up to 100% due to different sources of data. AR and X-linked deafness are usually more severe than autosomal-dominant forms (47). Most non-syndromic hearing losses are due to mutations in the connexin 26 gene (Cx26) (44, 48), accounting for about 50% of recessive hearing loss and about one-third of all genetic hearing loss. A second gene accounting for a large percentage of non-syndromic hearing loss is the cadherin 23 gene (49) (see below).

Because no deafness genes have definitively been identified for any form of deafness in dogs or cats, no equivalent gene-associated deafness statistics exist. When available, deafness prevalence data for different species and breeds are presented below.

## Naming of Genes and Forms of Deafness

Genes or gene loci are assigned names by the Human Genome Organization (HUGO) (50) (see Table 2 for resource web sites with hereditary deafness content). The Hereditary Hearing Loss Homepage (51) lists data and links for all known human deafness gene localizations and identifications for monogenic non-­syndromic hearing impairment, plus the most frequent monogenic syndromic deafness forms. Links are provided to their listing on the Online Mendelian Inheritance in Man (OMIM) web page (Table 2). The naming conventions are shown in Table 3 for non-syndromic genes and loci, syndromic genes and loci with their associated syndrome components, and mitochondrial genes and loci. As of 2012 nearly 125 loci for non-syndromic hearing loss had been discovered, with 54 being AD, 71 AR, 5 X-linked, two modifiers, and one Y-linked (12), and the numbers identified continue to increase at a rapid pace. The most frequent causative genes for AR hearing loss, in order of frequency, are *GJB2, SLC26A4, MYO15A, OTOF, CDH23*, and *TMC1*, with at least 20 mutations of each reported (12). The most common autosomal-dominant causative genes are *WFS1, MYO7A*, and *COCH*. Studies in mice have played an important role in these identifications since large numbers of induced mutations in mice can be quickly screened for deafness. Based on orthologous genes extracted from available complete genomes (OMA Browser, Table 2), the identified human genes provide a basis for identifying potential genes responsible for hereditary deafness in domestic animals and an understanding of the mechanisms by which different gene mutations result in deafness. Experimentally induced deafness-producing gene mutations in mice have in turn assisted in identifying human deafness gene mutations (3, 5, 22, 52).

**Table 2:** **Resource Web sites with hereditary deafness content**.

Web page name	URL
Hereditary Hearing Loss Homepage	http://hereditaryhearingloss.org/
Online Mendelian Inheritance in Man	http://omim.org/
Online Mendelian Inheritance in Animals	http://omia.angis.org.au/home/
Hereditary Hearing Impairment in Mice	http://hearingimpairment.jax.org/
Deafness Gene Mutation Database	http://hearing.harvard.edu/db/genelist.htm
Connexins and Deafness Homepage	http://davinci.crg.es/deafness/
HUGO Gene Nomenclature Committee	http://www.genenames.org/
OMA (“Orthologous MAtrix”) Browser	http://omabrowser.org/oma/home/
International Federation of Pigment Cell Societies – Color Genes	http://www.espcr.org/micemut/
International Mouse Phenotyping Consortium	https://www.mousephenotype.org/
Mouse Genome Informatics	http://www.informatics.jax.org/
HomoloGene	http://www.ncbi.nlm.nih.gov/homologene
Deafness in Dogs and Cats	http://www.lsu.edu/deafness/deaf.htm

**Table 3:** **Naming conventions for deafness genes and loci in humans**.

Non-syndromic genes and loci	
• Autosomal dominant (AD) – DFNA	
• Autosomal recessive (AR) – DFNB	
• X-linked – DFNX	
• Y-linked – DFNY	
• AUNA (auditory neuropathy autosomal dominant) – AUNA	

**Syndromic genes and loci**	**Syndrome components in addition to deafness**

• Alport – COL4A5	• X-linked or AR – collagenopathy, kidney disease, eye abnormalities
• Branchio-oto-renal – BOR	• AD – hypoplastic kidney and neck/preauricular pits or absence of pinna
• Jervell & Lange-Nielsen – JLNS	• AR – long QT syndrome
• Neurofibromatosis Type II – NFII	• AD – neural tissue tumors, pigmentation disorders
• Norrie – NDP	• X-linked recessive – blindness
• Pendred – PDS	• AR – goiter and hypothyroidism
• Stickler – STL	• AD – 2 or 3 forms – progressive arthro-ophthalmopathy, collagenopathy, facial abnormalities, joint problems
• Treacher Collins – TCOF or POLR	• AD – craniofacial abnormalities
• Usher – USH	• AR – 3 forms – progressive visual impairment from retinitis pigmentosa
• Waardenburg – WS	• AD or AR – 9 forms – pigmentation and neural crest defects

**Mitochondrial genes and loci**	

• Syndromic – MTTL	
• Non-syndromic – MTRN, MTTS	

**Figure 1:**
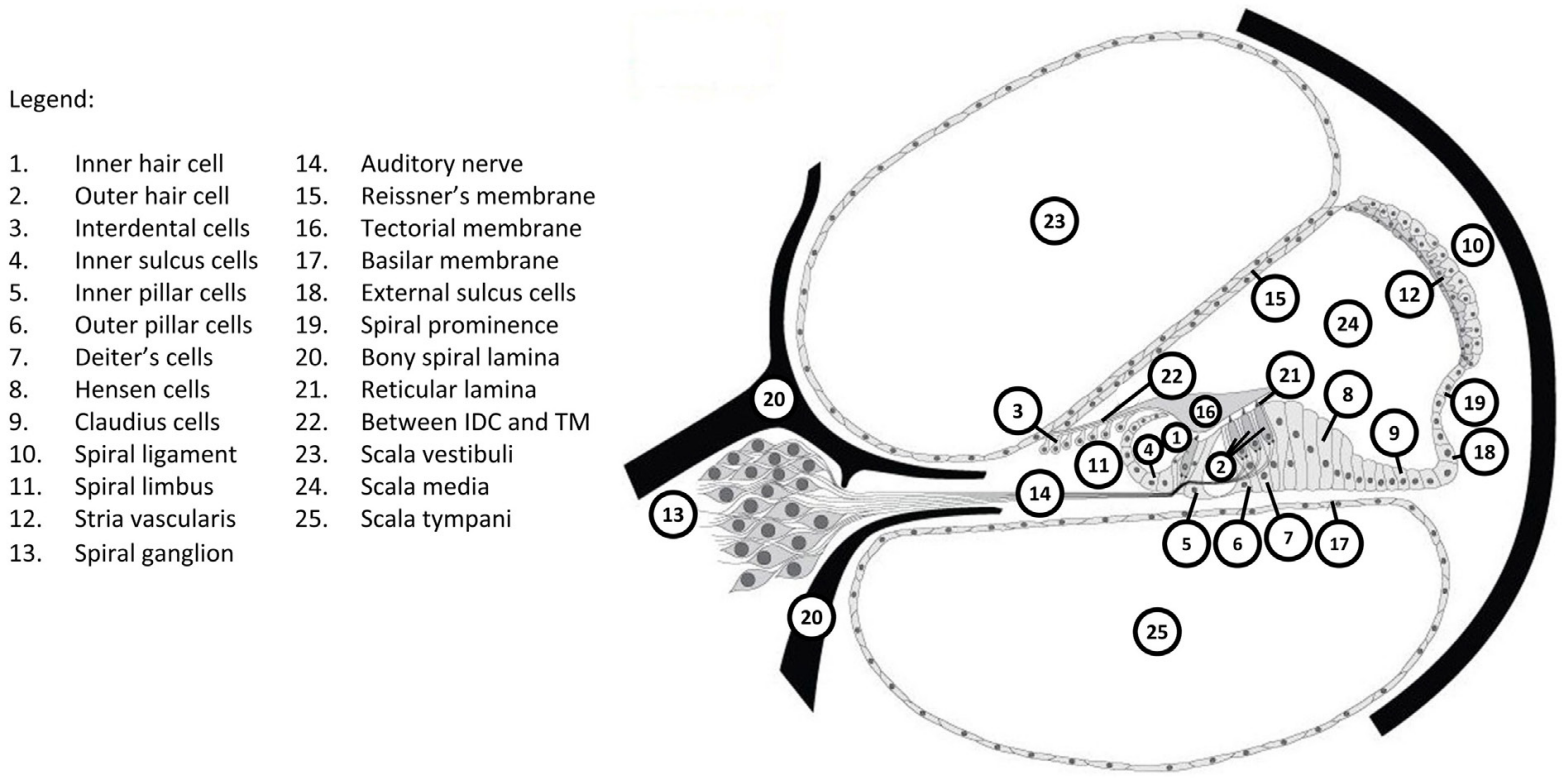
**Structures within the cochlea associated with the expression of genes identified to be causative for deafness**. *GJB2* is only expressed in regions 3–11 and 18. *CDH23* is only expressed in regions 1, 2, and 15. Reproduced with permission from Van Camp and Smith (51).

Identification of causative genes has led to the mapping of expression of these genes in the many different structures and tissues in the cochlea. Figure 1 shows the major components of the cochlea; the Hereditary Hearing Loss Homepage (51) provides a graphic demonstration of the expression of major deafness genes in these various structures.

As examples of the information available on identified deafness genes, two prominent examples of recessive deafness genes will be considered. One of the first identified genes responsible for human deafness was the gene for connexin 26 (48). Mutations of this gene account for 50% of AR hearing loss and 20–30% of all congenital hearing loss (44, 48, 53). Connexins are transmembrane transport channels for ions or small molecules that form gap junctions between cells that in the inner ear allow passage of potassium ions. The official name for the gene encoding the gap junction protein connexin 26 is *GJB2*, named for *gap junction protein beta-2* (OMIM entry 121011). The *GJB2* gene is expressed in cochlear regions 3–11 and 18 of Figure 1, including cells in the lateral wall, spinal limbus, and organ of Corti support cells, but not inner or outer hair cells (54). The mutation in *GJB2* identified by Kelsell et al. (48) results in a non-syndromic recessive deafness, which gave it the deafness identifier DFNB1 (OMIM entries 612645 and 220290). More than 110 different *GJB2* mutations causing hearing loss have been identified in humans (12), where the hearing loss can vary from profound to mild. More detail is available on the Connexins and Deafness Homepage (Table 2). A conditional knockout mouse mutation of *GJB2* has been reported (55) that has permitted following the progression of hearing loss with *GJB2* mutations.

Another prominent gene responsible for human recessive non-syndromic deafness is that for the protein cadherin 23 (*CDH23*, OMIM entry 605516) (49). Cadherins, named for calcium-dependent adhesion, are another group of cell adhesion transmembrane proteins that form calcium-dependent adherens junctions or belt desmosomes. The *CDH23* gene is expressed in inner and outer hair cells and Reissner’s membrane (regions 1, 2, and 15 of Figure 1) (56). A form of non-syndromic recessive deafness caused by mutations in *CDH23* is classified as DFNB12 (OMIM entry 601386), where the hearing loss is moderate to profound and progressive. Mutations of the mouse gene orthologous to *CDH23* produce the deaf mutant waltzer (v) line (57). Another *CDH23* mutation is responsible for Usher syndrome type 1D (USH1D).

## Deafness in Domestic Animal Species

An ultimate goal in the study of deafness in domestic species is to identify responsible gene mutations that can be used to develop DNA tests for use in individual animals to eliminate or reduce deafness prevalence. At present, few such genes have been identified, and the mechanism of inheritance – recessive, dominant, polygenic, etc. – may not even be known. As a result much of the discussion that follows will be based on classical (Mendelian) gene loci and phenotypes that have been shown to be linked to the disorders. The greatest number of hereditary deafness forms has been identified in dogs, followed by cat, horse, and a few other species. Although many of these deafness disorders are associated with pigmentation traits, none have been designated as syndromic since the pigmentation effects are not necessarily considered to be a phenotype abnormality or disease, and diseases of other tissues and systems are not present. However, since dogs homozygous for the merle locus are occasionally affected by both ocular disorders and deafness, this form of deafness could be argued to be syndromic.

Because the deafness expressions that occur in multiple breeds of a species are similar, they could be assumed to be the same disorder unless eventually demonstrated to be different. This assumption may not necessarily be borne out – for example, progressive retinal atrophy in different dog breeds results from several different gene defects – but it will require the identification and sequencing of the causative gene mutations in different breeds before a common mutation can be established or ruled out. The greatest prevalence of hereditary deafness in all domestic species is cochleo-saccular (CS), associated with the genes responsible for white pigmentation.

The web site Online Mendelian Inheritance in Animals (Table 2) identifies nine records for deafness: *Canis lupus familiaris* (dog) (OMIA 000259-9615), *Equus caballus* (horse) (OMIA 000259-9796), *Felis catus* (domestic cat) (OMIA 000259-9685), *Lama glama* (llama) (OMIA 000259-9844), *Neovison vison* (American mink) (OMIA 000259-452646), *Sus scrofa domesticus* (domestic pig) (OMIA 000259-9825), *Vicugna pacos* (alpaca) (OMIA 000259-30538), *Canis lupus familiaris* (dog, adult-onset) (OMIA 001727-9615), and *Bos taurus* (cattle) (OMIA 001680-9913). These and others are discussed below.

### Dogs

Hereditary deafness in dogs can be (1) congenital or late onset, (2) CS, neuroepithelial, or other, and (3) sensorineural or possibly conductive.

#### Canine Congenital Deafness

##### Pigment-associated cochleo-saccular deafness

Many of the recognized forms of hereditary deafness in multiple species are associated with white pigmentation, where the pathology is CS. The recessive alleles of the piebald locus and the dominant allele of the merle locus are associated with congenital hereditary deafness in dogs, while the dominant allele of the white locus is associated with deafness in cats. The genetics of coat color pigmentation in dogs and cats have been reviewed elsewhere (58–60); a partial listing is shown in Table 4. Only relevant pigment loci will be considered here, including loci important in pigmentation patterns such as *Brown* (*B*) and *Ticking* (*T*) that are not linked to deafness but that nevertheless play an important role in animal phenotypes. Discussion is presented in terms of the pigment loci rather than specific dog breeds because of the wide acceptance and demonstration of the association between deafness and those loci.

**Table 4:** **Major Mendelian pigmentation loci and associated genes and phenotypes in dogs and cats**.

Locus/category	Gene	Allele^a^	Phenotype
**DOG**			
**Spotting**			
*S* (Spotting)	*MITF*		
		*S*	Solid coat (no spotting, wild type)
		*s^i^*	Irish spotting pattern
		*s^p^*	Piebald spotting pattern
		*s^w^*	Extreme white spotting pattern
*T* (Ticking)	Unknown		
		*T*	Ticking in white areas
		*t*	No ticking (wild type)
*M* (Merle)	*PMEL*		
		*M*	Merle pattern
		*m*	Non-merle (wild type)
**Dilution**			
*B* (Brown)	*TYRP1*		
		*B*	Black – no dilution of eumelanin (wild type)
		*b*	Liver, brown, chocolate – diluted eumelanin
*C* (Albino)	Unknown		
		*C*	No dilution of pheomelanin (yellow, sable, fawn, wild type)
		*c^ch^*	Dilution of pheomelanin (white, cream)
*H* (Harlequin)	*PSMB7*		
		*H*	Harlequin pattern (in a merle background)
		*h*	Merle pattern (in a merle background)
*Tw* (Tweed)	Unknown		
		*Tw*	Large smooth patches (in a merle background)
		*tw*	Small, jagged patches (in a merle background, wild type)
**Pigment-type switching**			
*A* (Agouti)	*ASIP*		
		*A^y^*	Yellow, sable, fawn
		*a^w^*	Agouti-banded hair, light colored ventrum
		*a^t^*	Black-and-tan
		*a*	Recessive black
*K* (Kurokami)	*CBD103*		
		*K^B^*	Black
		*k^br^*	Brindle (black and yellow stripes)
		*k^y^*	Wild type (allows expression of Agouti phenotypes)
*E* (Extension)	*MC1R*		
		*E^m^*	Melanistic mask
		*E*	Extension, wild type
		*e*	Recessive yellow
**CAT**			
**Spotting**			
*W* (White)	*KIT*		
		*W*	White coat
		*W^h^*	High degree of spotting
		*W^l^*	Low degree of spotting
		*w*	Solid (wild type)
**Dilution**			
*B* (Brown)	*TYRP1*		
		*B*	No dilution of eumelanin (black, wild type)
		*b*	Chocolate
		*b^1^*	Cinnamon
*C* (Albino)	*TYR*		
		*C*	No dilution of melanin (yellow, sable, fawn)
		*c^b^*	Burmese (temperature-sensitive dilution pattern, pointed coat)
		*c^s^*	Siamese (temperature-sensitive dilution pattern, extreme pointed coat)
		*c*	Albino
**Pigment-type switching**			
*A*(Agouti)	*ASIP*		
		*A*	Agouti-banded hair (wild type)
		*a*	Recessive black
*E* (Extension)	*MC1R*		
		*E*	Extension (wild type)
		*e*	Amber, age-dependent fading of tabby pattern

*Modified from Schmutz and Berryere (59) and Kaelin and Barsh (60)*.

*^a^Alleles listed in order of dominance*.

###### Genetics and regulation of pigmentation

Much has been written about the complex genetics of melanocytes and the pigmentation pathway (61–68). A brief consideration of selected aspects is presented below. More than 125 distinct genes are known to directly or indirectly regulate pigmentation (69). During embryological development, melanoblasts from the neural crest migrate to target sites (skin, hair, eyes, stria vascularis), differentiate into melanocytes, and then produce melanins as appropriate; more than 25 genes are involved in these processes (68). Melanocytes produce two major types of melanins: eumelanin, responsible for black, brown, and blue color; and pheomelanin, associated with red, yellow, and cream color (59). A major mechanism in pigment expression in the skin is the G protein-coupled receptor melanocortin 1 receptor (MC1R). The agonists α-melanocyte-stimulating hormone (αMSH) and adrenocorticotropic hormone (ACTH) activate MC1R to produce eumelanin, while the MC1R antagonist agouti signaling protein (ASP) causes melanocytes to produce pheomelanin. Endothelin 3 acts through its receptor endothelin receptor type B (EDNRB) on melanocytes to enhance expression of MC1R. The gene for MC1R is a major source for melanoma development, and has been shown to be the Mendelian pigment locus *E* (Extension) that is responsible for the production of a melanistic mask or the expression of recessive yellow (Table 4) (60).

**Figure 2:**
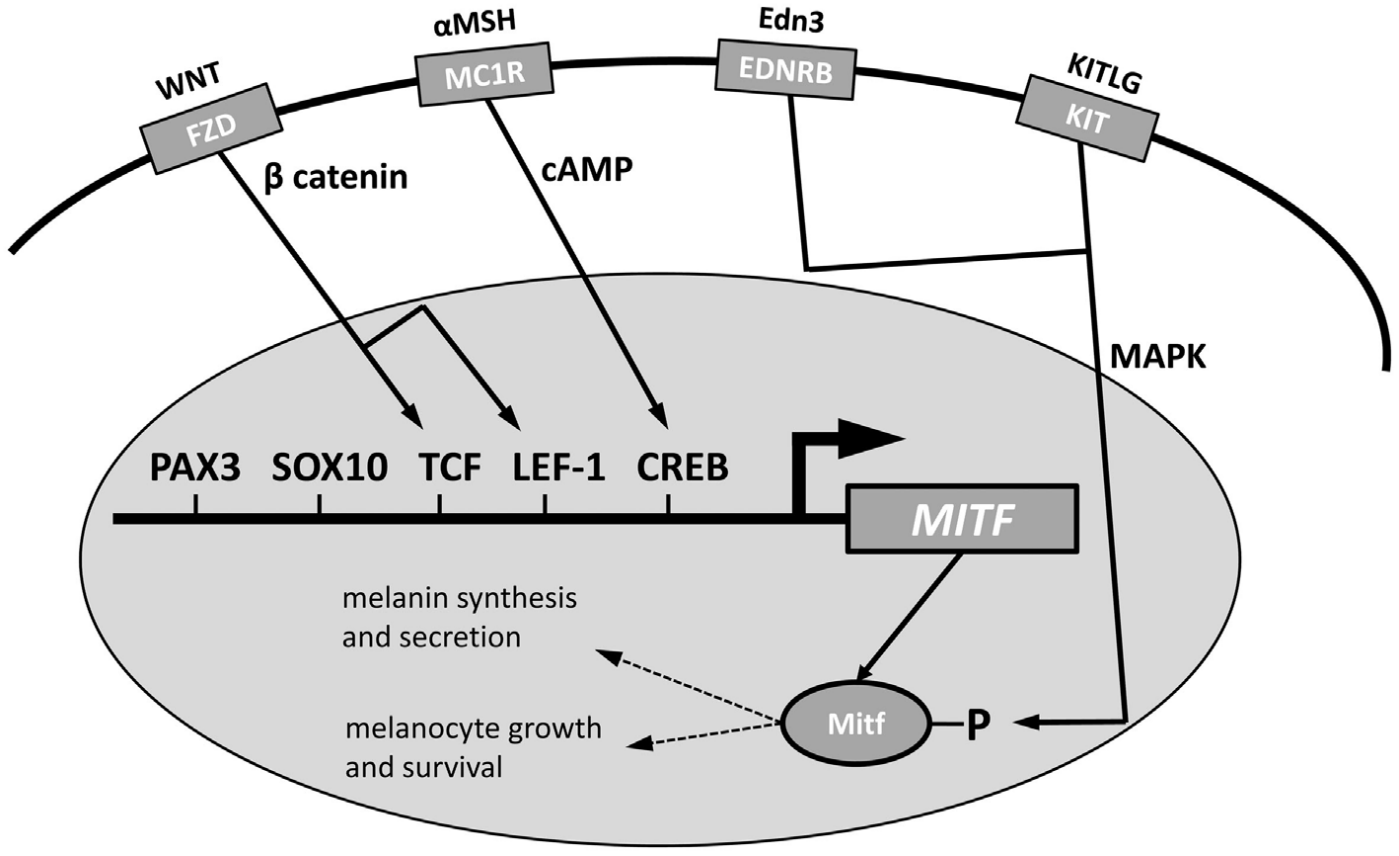
**Modulation of *MITF* function in the nucleus of a melanosome to activate melanin synthesis and secretion and melanocyte growth and survival**. The promoter region of *MITF* can be modified by *PAX3, SOX10, TCF, LEF-1*, and *CREB*; *TCF* and *LEF-1* are activated by β catenin from Wnt activation of the *FZD* receptor and *CREB* is activated by cAMP from *MC1R* activation by αMSH. *EDNRB*, activated by Edn3 and *KIT* activated by KITLG, act on the MAPK pathway to influence posttranslational *Mitf* function. Modified from Price and Fisher (63).

Multiple genes in the pigmentation pathway interact with microphthalmia-associated transcription factor (*MITF*), the master transcriptional regulator of pigmentation (63, 67, 70) (Figure 2). *MITF* is necessary for melanocyte proliferation and development. The *MITF* promoter is affected by the transcription factors *PAX3, SOX10*, *TCF, LEF-1*, and *CREB* (63). These genes are affected by upstream signaling pathways. The Frizzled (FZD) receptor, activated by Wnt, produces β catenin, which acts on TCF and LEF-1 to promote melanocyte differentiation. αMSH acts on the MC1R receptor to produce cAMP to act on CREB. Posttranslational effects on MITF come from several signaling pathways, including the MAP kinase (MAPK) pathway that is activated by binding of Endothelin 3 to the EDNRB or by activation of the proto-oncogene Kit receptor by stem cell factor (Scf, KITLG).

Melanogenic proteins downstream from *MITF* include Pmel17, MART-1, TYR, TYRP1, and Dct. The enzymes tyrosinase (TYR), TYRP1, and Dct affect the quantity and quality of synthesized melanins, while the proteins Pmel17 and MART-1 are required for structural maturation of melanosomes (68, 71). TYR is the rate-limiting enzyme in the synthesis of melanin, hydroxylating tyrosine to L-3,4-dihydroxyphenylalanine (DOPA), which is subsequently converted to eumelanin or pheomelanin.

Mutations of TYR produce albinism, the condition where melanocytes are present but produce no melanin. Only pigmented tissues are affected. Multiple types of albinism/hypopigmentation have been described in humans (68, 72). There is no association between albinism and hearing loss, since melanocytes are present in the stria vascularis of albinos, but melanin is not produced, and albinos do not exhibit hereditary deafness. As a result, a secondary role of *MITF* is implicated. The stria is responsible for maintaining the endocochlear potential, a potential of approximately +100 mV in the cochlear duct critical for survival of hair cells, and evidence indicates that melanocytes are responsible for production of the endocochlear potential (73).

Because of the complexity of the genes and signaling pathways affecting pigmentation, and the number of mutation targets, the identification of a gene responsible for pigment-associated deafness is no simple task.

###### Piebald locus (*S*)

The white spotting locus *S* has four alleles: the dominant allele *S* produces solid color, determined by other loci, while the recessive alleles express increasing amounts of white in the coat: Irish spotting (*s^i^*), piebald (*s^p^*), and extreme white piebald (*s^w^*) (Table 4). Because melanocytes must migrate from the neural crest during embryogenesis, the body regions most distant from the dorsal midline are the most likely to be unpigmented/white: the feet, chest, muzzle, and tail. Examples of breeds with Irish spotting are Basenji, Australian shepherd, border collie, and Bernese mountain dog. Examples of breeds with piebald are English springer spaniel, fox terrier, whippet, Bracco Italiano, and beagle. Examples of breeds with extreme white piebald are Dalmatian, bull terrier, Staffordshire terrier, and Samoyed. Because the alleles are recessive, white dogs must be homozygous for a recessive allele, but it is not known how often dog breeds mix two different recessive alleles. The boxer Tasha that was used to sequence the canine genome had a phenotype designated as flash (*Ss^w^*), which enabled construction of BACs (bacterial artificial chromosomes) for each allele from her DNA (74). Significant associations between deafness and the recessive piebald alleles have been demonstrated for Dalmatian, English setter, and English cocker spaniel (75), border collie (76, 77), Australian cattle dog (78), and Jack Russell terrier (79). It has also been shown that a strong relationship exists between deafness and the absence of pigment in the iris and tapetum lucidum, interpreted to be evidence of strong expression of the piebald locus (75). Likewise, a significant negative relationship has been shown between deafness and patch in Dalmatians (75) and double masks and body patches in Australian cattle dogs (78), interpreted to be evidence of weak expression of the piebald locus. Among breeds where data are available, the prevalence of deafness in dog breeds with the recessive piebald alleles is highest for the Dalmatian, where 8% of US dogs are bilaterally deaf and 22% are unilaterally deaf; prevalence rates are lower in countries that do not allow blue eyes in the breed standard (75). Other breeds with high prevalence rates include Australian cattle dog, bull terrier, English setter, English cocker spaniel, Catahoula leopard dog, Jack Russell terrier, Boston terrier, and Dogo Argentino (80).

White spotting is the result of an absence of melanocytes in skin and hair follicles from a failure of melanocyte migration, proliferation, or survival during development (73). The white spotting locus or piebald locus is colocalized with the *MITF* gene, located on canine chromosome 20 (CFA20) (OMIM entry 156845) (81–83). *MITF* regulates the differentiation, migration, and survival of neural crest-derived melanocytes during development, including melanocytes involved in coat color and melanocytes in the cochlear stria vascularis. *MITF* also regulates the gene *tyrosinase* (*TYR*, OMIM entry 606933), which encodes for the rate-limiting enzyme in the synthesis of melanin pigment. Two *MITF* mutations, located in a 3.5-kb region upstream of the M promoter region, were proposed by Karlsson et al. (82) in different combinations to explain the three recessive white spotting alleles. The first was a 198-bp short interspersed element (SINE) insertion located 3,167 bp before the start codon of exon 1M, and the second was a polymorphism in the gene promoter region. The SINE insertion was present in *s^p^* and *s^w^* dogs, but not in *s^i^* or *S* dogs. The length polymorphism that was present in *S* dogs was longer and differed slightly in *s^i^*, *s^p^* and *s^w^* dogs. The exception was the Dalmatian, where the polymorphism resembled that of solids.

Schmutz et al. (84) studied one of the six known isoforms of *MITF* specifically expressed in neural crest-derived cells, known as *MITF-M* (85). As had been reported by Karlsson et al. (82), none of the dogs from five breeds with Irish spotting had the SINE insertion in *MITF-M*, but dogs from 19 breeds with random white spotting (piebald or extreme white) were homozygous for the insertion. Most heterozygotes for the SINE were either solid or had minimal white markings, but some had white undersides with a white collar. In contrast to Karlsson et al. (82), Schmutz et al. (84) were unable to find evidence for either a specific *s^w^* allele at *MITF-M* or one for *s^i^*. They also were unable to replicate the length polymorphism described by Karlsson et al. (82). A suggestion has been made that *KITLG* is a candidate gene for *s^i^* (86), but there have been no further reports on this possibility.

Subsequent to the Karlsson et al. (82) study, a recent publication from the same lab (74) confirmed that the SINE insertion is present in *s^p^* and *s^w^* dogs but not in *S* and *s^i^* dogs, and further concluded that the three recessive alleles were not due to independent mutations but instead to haplotype effects from combinations of different length polymorphisms. The authors stated that “*MITF* in dogs is another example of ‘evolution of alleles’ by consecutive accumulation of multiple causal mutations,” in this case in the length polymorphism in the *MITF-M* promoter. Findings in the Dalmatian were again different from other dogs with recessive alleles, with a length polymorphism shorter than those found in other spotted dogs but still longer than those in *S* dogs. The effects of the length polymorphism on the recessive alleles is to affect *MITF-M* transcription by alteration of the DNA helix to modify binding sites for LEF-1, SOX10, and PAX3 that are critical for *MITF-M* expression, and subsequent melanocyte function. *PAX3* (*paired box gene 3*, OMIM entry 606597) is a paired box gene, formerly known as *splotch*, associated with eye, ear, and face development. *SOX10* (*sry-box 10*, OMIM entry 602229) is a homeobox gene regulated by *MITF* that is involved in neural crest and peripheral nervous system development. Mutations of *PAX3* and *SOX10* are responsible for deafness in several forms of human Waardenburg syndrome. *LEF-1* (*lymphoid enhancer-binding factor 1*, OMIM entry 153245) is involved in transcriptional self-activation of the *MITF-M* promoter, while PAX3 and SOX10 are transcription factors critical for *MITF-M* transcription. It was suggested that the SINE acted as a weak silencer element acting in addition to the length polymorphisms to reduce the *MITF-M* promoter activity associated with *s^p^* and *s^w^* (74).

The molecular genetics of the *S* alleles still require more elucidation. Commercially available DNA tests for “piebald” apparently only test for *s^p^* and *s^w^* dogs, based on the presence of the SINE insertion, but due to the lack of consensus on whether *s^w^* exists, the test may only demonstrate the presence of *s^p^*. Most commercial pigmentation tests evaluate other pigmentation loci (87).

No mutations in the SINE or length polymorphism have been demonstrated to co-segregate with deafness. These results, along with the positive association between deafness and blue eyes and the negative association between deafness and patches in Dalmatians, and double masks and body patches in Australian cattle dogs, suggest that a polygenic mechanism may explain deafness, with interactions between *MITF-M* and another modifying gene determining hearing status in white-spotted dogs. Interestingly, humans with blue eyes may be more susceptible to noise-induced hearing loss than people with darker eyes (88, 89).

Separate from the question of causative gene mutations responsible for deafness is the question of the mechanism of inheritance of pigment-associated deafness (90), knowledge that would be helpful in reducing disease prevalence. This is complicated by variable disease expression, since dogs can be deaf in either one or both ears and it is possible that dogs can be genotypically deaf but not exhibit the phenotype. Most attempts to establish an inheritance mechanism have utilized complex segregation analysis to analyze multigenerational hearing pedigrees to identify a major segregating locus with a large effect on the expression of deafness. In Dalmatians, Famula et al. (91) concluded that a single allele affected the prevalence of deafness but that one locus could not completely explain the inheritance. Muhle et al. (92) concluded that their data were best fit by a single locus with an incompletely penetrant recessive allele. Juraschko et al. (93) presented evidence for a monogenic–polygenic model with a recessive major gene, and Cargill et al. (94) reported that evidence was not persuasive for a single major gene affecting deafness. Analyses of Jack Russell terrier data found that a single locus model of inheritance was also not supported (95). Sommerlad et al. (96) reported that deafness in Australian stumpy-tail cattle dogs followed an AR pattern. Both Jack Russell terriers and Australian stumpy-tail cattle dogs carry recessive *S* alleles.

Studies have been conducted to identify loci of genetic mutations responsible for deafness using a variety of molecular genetic techniques. Rak et al. (97) mapped 20 candidate genes for deafness in the dog that have been identified in other species, but none were informative. Cargill (98) utilized the MSS1 set of 172 microsatellite markers for whole-genome screens of Dalmatian DNA (99). Maximum LOD scores for deafness were found with markers Cos15 on CFA17 (LOD = 1.69) and FH2585 on CFA28 (LOD = 1.34), but the LOD scores did not reach the significance level. One human deafness gene is located on the human marker HSA02, syntenic to CFA17. Within this region is the deafness locus for DFNB9, a non-syndromic deafness locus caused by a recessive mutation in the gene *Otoferlin* (*OTOF*, OMIM entry 603681) (100). However, with the possibility of a polygenic mechanism for deafness, a LOD score well above 3 would be necessary for significant association.

Stritzel et al. (101) performed an association study using a set of eight markers, two flanking and six internal to *MITF* in Dalmatians to identify associations between the markers, deafness, and blue irises. After genotyping, only four markers were polygenic in the Dalmatian subjects and therefore used for the association analysis. The authors concluded that *MITF* is involved in deafness in Dalmatians and that possible causative mutation or mutations might be in the non-coding sequence of *MITF*, but no actual gene mutation was identified or suggested to be a candidate.

Sommerlad et al. (96) performed whole-genome microsatellite studies of Australian stumpy-tail cattle dogs and found a significant linkage (LOD = 3.64) with a locus on CFA10. A candidate pigmentation and deafness gene located within the locus was *Sox10*; however, sequencing of the gene in six hearing, two unilaterally deaf, and two bilaterally deaf dogs did not demonstrate any deafness-associated *Sox10* mutations.

Kluth and Distl (102) performed a genome-wide association study in Dalmatians using the Illumina canine bead chip, which contains 173,662 single nucleotide polymorphism markers (SNPs) spanning the canine genome, to identify quantitative trait loci (QTL) associated with deafness. Using a mixed linear model of DNA from all deaf dogs, significant loci were found on CFA2, 6, 14, 17, 27, 29, and 31. Affected dogs were also segregated by eye color since blue-eyed dogs are significantly more likely to be deaf than brown-eyed dogs. Six significant loci associated with deafness were identified in brown-eyed Dalmatians (CFA2, 6 (two), 14, 27, and 29) and four significant loci were found in blue-eyed Dalmatians (CFA6, 14, 27, 29, and 31), but only the locus on CFA27 overlapped both groups. The authors concluded that several loci contribute to pigment-associated hereditary deafness in Dalmatians. Overall, significant loci found on CFA6, 14, 27, 29, and 31 were located in close proximity to genes shown to be causative for hearing loss in humans or mice: *COL11A1* (*collagen type XI alpha 1*) on CFA6, *DFNA5* and *HOXA1* (*homeobox A1*) on CFA14, *GDAP1* (*ganglioside-induced differentiation associated protein 1*) on CFA29, and *CLDN14* (*claudin-14*) on CFA31. These genes are involved in the development and maintenance of inner ear structures. Genes associated with melanocyte function were located in close proximity to four of the significant loci: *ARHGAP12* (*Rho GTPase activating protein 12*) on CFA2, *TWF1* [*twinfilin, actin-binding protein, homolog 1* (*Drosophila*)] on CFA27, *CDC42EP2* on CFA18, and *AEBP2* (*AE* (*adipocyte enhancer*) *binding protein 2*) on CFA27. Two additional genes located in close proximity of significant loci may also have associations with auditory function: *GIPC2* (*GIPC PDZ domain containing family, member 2*) on CFA6 and *CRIM1* (*cysteine rich transmembrane BMP regulator 1* (*chordin-like*)) on CFA17. See Kluth and Distl (102) for discussion of the functions of these genes. No significant loci were located within or near the genes *MITF*, *SILV*, *SOX10*, or *KITLG*, shown in other studies to be associated with deafness (82, 96, 101, 103–105). A marker near *MITF* previously shown to have an association with deafness in German Dalmatians (101) did not reach significance in this study.

In summary, a wide variety of candidate genes have been investigated as causative for deafness in *S* locus dogs, but no consensus gene or genes have yet been established.

###### Merle locus (*M*)

The *M* locus has two alleles: the recessive allele *m* produces uniform pigmentation, determined by other loci, and the dominant allele *M*, known as merle or dapple, which produces a random pattern of diluted pigmentation overlying uniform pigmentation; it also increases the amount of white spotting on the coat (Table 4). Breeds that may carry the dominant merle allele include Australian shepherd, Shetland sheepdog, Catahoula, Cardigan Welsh corgi, Dachshund, Great Dane, Chihuahua, American pit bull terrier, American Staffordshire terrier, Beauceron, border collie, Koolie, poodle, Pyrenean shepherd, Old English sheepdog, American cocker, Pomeranian, Hungarian Mudi, and Norwegian Dunkerhound among others. Dogs homozygous for the dominant allele can be deaf and frequently have ocular abnormalities (106), and heterozygous dogs can be deaf, but there may be breed differences in the prevalence of deafness in merle carriers (107). In general, the prevalence of deafness in dogs carrying merle is similar to that of breeds with piebald, although merle-carrying breeds were long considered to be tainted with a high deafness prevalence based on a flawed study in Dachshunds (108); see Strain et al. (107) for discussion. Some breeds, such as Great Danes, carry both merle and piebald, and some breeds with merle, such as Great Danes, also carry dominant dilution loci known as harlequin (*H*) and tweed (*Tw*) that modify merle (Table 4) (109–111), but no association with deafness has been shown for either *H* or *Tw*. As with piebald, blue eyes in merle dogs may be associated with deafness.

The merle gene has been sequenced and shown to be a mutation in the dominant allele of the *PMEL* (*premelanosomal protein*) (OMIM entry 155550) pigmentation gene (previously known as *SILV* or *silver*), located on canine chromosome 10 (CFA10) (104, 112, 113). This gene encodes a melanocyte-specific type I transmembrane glycoprotein (Pmel17) that is important in the structural organization of premelanosomes. The mutation in *PMEL* is a 253-bp SINE retrotransposon insertion located at the boundary between intron 10 and exon 11 in the gene, and includes a multiple adenine repeat (poly-A) tail that must be 90 to 100 adenine repeats in length to cause expression of the merle phenotype. Shorter (<65) poly-A tails produce “cryptic” merles that do not have the merle phenotype but produce merle offspring.

An example of the cryptic pattern was illustrated by genotyping of two Australian shepherds (107). A genotyped male was *Mm*, while its genotyped daughter was *MM*. The daughter’s dam (not genotyped) was a black tricolor without the merle pattern. The daughter produced both merle and tricolor phenotypes when bred (not genotyped). As a result, one of the dominant merle alleles in the daughter had to be cryptic with a truncated poly-A tail (*M’*) and the tricolors out of the daughter had to carry the cryptic merle allele. The mother had to also carry the cryptic merle allele to produce a daughter with a *MM* genome (actually *M’M*). A heterozygous cryptic merle (*M’m*) bred to a normal heterozygous merle (*Mm*) can produce heterozygous cryptic merles (*M’m*), heterozygous normal merles (*Mm*), non-merles (*mm*), and homozygous merles (*M’M*). Only the *Mm* and *M’M* outcomes will show the merle phenotype. Without genotyping, studies of dogs carrying merle can lead to incorrect conclusions. A DNA test is now available to identify heterozygous (*Mm*) or homozygous (*MM*) carriers of the dominant allele of the merle gene.

Sequencing for defects in merle in deaf dogs has shown no mutations in the gene, so there is no explanation for the association between the pigmentation gene and deafness, and the mechanism of inheritance of the deafness is unknown.

###### White (*KIT*), kit ligand, and endothelin receptor type B loci

Another white spotting locus in many species is dominant white *W*, caused the proto-oncogene *KIT*, but it has not been considered to play a role in the dog. *KIT* is responsible for white spotting patterns in pig, cow, horse, cat, and human (114). *KIT* is involved in neural crest cell migration and differentiation into melanoblasts. There appears to be a reciprocal interaction between *KIT* and *MITF*, in that *MITF* is necessary for *KIT* expression in melanoblasts and *KIT* signaling modulates *MITF* activity and stability in melanocytes (62). Mutations of *Kit* in the mouse and other species can produce deafness (115). *KIT* is the gene for the tyrosine kinase receptor (OMIM entry 164920) acted on by its ligand (*KIT Ligand*, *KITLG*, OMIM entry 184745); *KITLG* is also known as *mast cell growth factor (MGF) stem cell factor* or *Scf*.

Tsai et al. (116) sequenced *KIT* in a deaf Dalmatian and a hearing Labrador retriever, with location on CFA13. There was 100% identity in the sequences of the two dogs, suggesting that *KIT* plays no role in deafness in Dalmatians. van Hagen et al. (117) evaluated *KIT* and *EDNRB* to better understand white spotting in boxers, but found no association with the genes and white spotting; hearing status was not considered, but there is a high prevalence of deafness in white boxers. *EDNRB* (OMIM entry 131244) is acted on by endothelin 3 to act on the MAPK pathway to act on posttranslational *Mitf* (Figure 2), and *EDNRB* mutations are associated with lethal white foal syndrome in horses (118) and deafness in American Paint horses (119). Another study excluded *KIT* and *EDNRB* as the basis for white spotting in border collies (120). *KITLG*, located on CFA15, was excluded as the cause for merle (121).

A loss-of-function mutation in *KIT* was identified in a kindred of German shepherds that produced a white spotting pattern on the face, ventral abdomen, feet, and tip of the tail (122). The cause was determined to be a mutation in *KIT*, but all affected dogs had normal hearing based on brainstem auditory-evoked response (BAER) testing (123, 124). A similar *de novo* mutation in *KIT* was reported in a Weimaraner (125); hearing status for the dog was not reported.

###### Brown locus (*B*)

The Mendelian locus designated *B* in dogs has two alleles: a dominant allele *B* that produces black by preventing dilution of eumelanin and a recessive allele *b* that produces brown, liver, or chocolate by diluting eumelanin. The gene responsible for *B* is *tyrosinase-related protein 1* (*TYRP1*, OMIM entry 115501), shown by linkage analysis to be located on CFA11 (126). The protein product TYRP1 is involved in melanin synthesis, stabilizing tyrosinase; maintenance of melanosome structure; and affects melanocyte proliferation. *TYRP1* expression is regulated by *MITF*. Three variants were identified in the gene that was associated with the recessive allele: a premature stop codon in exon 5, a deleted proline residue in exon 5, and a less common variant in exon 2 (126). Based on nose leather color, all 43 brown dogs carried two or more of the variants, while none of 34 black dogs carried two or more variants (10 carried one variant). There is no known association between *B* and deafness. Black coloration in dogs can also result from the pigment-type switching locus *K* (*Kurokami*) (127, 128), which also has no known association with deafness.

###### Ticking locus (*T*)

Ticking (*T*) is a modifier of *S* in dogs, demonstrated in dogs with the dominant allele by pigmented spots or areas of skin and hair in regions that would otherwise be white. It has been suggested that ticking may be a second wave of melanocyte precursor differentiation, proliferation, or colonization of hair follicles, since that pattern does not appear until several weeks after birth (60). Dalmatians may be *TT* (129).

The pigmentation pattern in the Dalmatian is determined by three Mendelian loci: *B/b* (black or liver base coat color (130), *s^w^* (white overlay of base coat color), and *T* (provides holes in the white to display the base coat color). Early lore on deafness in the breed posited deafness prevalence differences based on spot size or heaviness of spotting, but this is determined by *T*, which has no relationship to deafness. There is no deafness prevalence difference between black (*B*) or liver (*b*) or even lemon (*ee*, extension pigment locus, Table 4, *MC1R* gene) spotted dogs, nor between males and females (131). The only significant associations between deafness and phenotype were a negative association for the presence of a patch and positive associations for absent pigment in the iris (blue eyes) and absent pigment in the tapetum lucidum behind the retina. Dalmatian spots do not appear until 3–4 weeks of age with the expression of *T*, but patches, which are larger than typical spots, are present a birth. We have suggested that a patch represents weak expression of *s^w^*, failing to cover the underlying base color, while blue eyes, absent tapetal pigmentation, and absent strial melanocytes represent strong expression of *s^w^*, possibly through the action of a gene that modifies piebald. A similar pattern occurs in Australian cattle dogs that are also white at birth. Dogs with equivalents of the Dalmatian patch – bilateral melanistic facial masks or body marks – are less likely to be deaf (78).

##### Flat-coated retriever

The flat-coated retriever derives from the same background as the Labrador retriever, and can be black, liver, or yellow; yellow is a disqualifying color. The colors parallel the black, liver, and lemon colors of the Dalmatian and probably have the same Mendelian loci bases.

Although deafness has been reported to occur in the breed,^1^ it does not appear to have been studied. In one US pedigree, the breeding of two colored flat coats produced one male and one female bilaterally deaf puppy. The sire was bred to a different female, and a hearing female offspring of that breeding was bred using 20-year old frozen semen from another male, producing two bilaterally deaf male puppies. The deafness appeared to follow an AR pattern, but further pedigree information was not available and no genomic studies were performed.

###### Inheritance of pigment-associated deafness

The inheritance of pigment-associated deafness seems complex and does not parallel the inheritance of the most commonly associated pigment loci merle (simple dominant) and piebald (simple recessive). Numerous studies have attempted to identify the mechanism of inheritance of deafness using complex segregation analysis of pedigrees or DNA microsatellite markers or SNPs from affected dogs, without a resulting consensus (90–96, 132); most proposed some version of an AR mechanism, but not one that is simple Mendelian AR. A number of candidate genes have been investigated but no clarity exists at present. Much work remains to solve the genetic basis of this form of deafness. Until DNA-based tests become available to identify dogs carrying gene mutations responsible for hereditary deafness, the best advice for breeders is that affected dogs should not be bred, even those deaf in just one ear, where the genetic defect is present but incompletely expressed.

##### Neuroepithelial deafness

Congenital deafness that is not associated with white pigmentation is less common among dogs and even less so in other domestic species. This discussion is presented based on the breeds affected, rather than specific gene loci.

###### Doberman pinscher

Bilateral congenital deafness accompanied by vestibular disease in Doberman pinschers was first reported by Chrisman (133) in the US and Skerritt (134) in England and analyzed in detail by Wilkes and Palmer (135). The disorder has a neuroepithelial cochlear pathology and has an AR mode of inheritance based on pedigree analysis (135). Cochlear hair cells degenerated, but the stria vascularis was intact and the only vestibular pathology was absent or abnormal otoconia in some affected animals. Vestibular signs of head tilt, ataxia, and circling behavior and rarely a spontaneous horizontal nystagmus develop between birth and 12 weeks of age, but the animals adapt to them with time; the deafness is permanent (133). Chrisman reported that not all dogs appeared to be deaf, but in a more detailed study of 21 affected dogs, all demonstrated deafness by BAER testing (135). Similar presentations have been reported in the beagle, Akita, German shepherd (136), and possibly in the Tibetan terrier (137) and Shropshire terrier (25, 132), and in several cat breeds (133, 136). Congenital vestibular disease has been reported in English cocker spaniels (138) but the dogs were not deaf.

A non-peer reviewed media source (139) has reported that a genetic cause for the disorder, which is said to be AR and to affect 13% of Doberman puppies, has been identified, but the gene identity and location have not been reported. Affected dogs are referred to as “dings” among breeders, and the deafness vestibular disease has been named DVDob.^2^ Oculocutaneous albinism not associated with deafness has been reported in the breed (140).

###### Puli

Deafness is recognized within breeders of the Puli, but no studies of it have been reported. In addition to the commonly recognized black coat, the breed standard also accepts solid colors of rusty black, all shades of gray, and white. Deafness occurs in black Pulis, so the pathology is likely NE, although black dogs could still carry the recessive *S* allele.

###### Labrador retriever

Congenital deafness with vestibular signs has been identified by the author in a small number of Labrador retrievers from several distinct lines. Three litters from a northeastern US state, sired by the same male to females from unrelated lines, each produced a deaf puppy, one unilaterally deaf and two bilaterally deaf. The stud dog was later sold to a kennel outside of the US. All affected dogs adapted to their vestibular signs. Four other Labradors out of two litters sired by one male to two unrelated females from a southern US state had vestibular signs (assessed by the referring veterinarian) and were BAER tested; two were 11 weeks old and two were 4 weeks old. Only one of the four was deaf (bilaterally) and all vestibular signs had resolved at the time of testing.

Histopathology from affected deaf puppies has not been assessed, but the prevalence seen to date suggests an AR inheritance. A histopathological study has been reported for a deaf Rottweiler puppy from unregistered parents which was actually 25% Labrador (26), in which the pathology was of the neuroepithelial pattern. Deafness in Labrador retrievers has been reported before the 1990s, but studies of it are not known to have been undertaken and no details are known.

#### Canine Late Onset Deafness

The existence of late onset hereditary deafness in dogs has only recently been recognized. Late onset sensorineural deafness has been reported in three breeds, and a form of late onset conductive deafness in another breed may have a hereditary component.

##### Nervous pointers

A champion field trial pointer bitch reputed to have experienced a nervous breakdown was used as foundation breeding stock to develop a line of dogs with enhanced anxious behavior to support research in human anxiety disorders (141–144). Concurrent with the breeding for increased anxious behavior was the appearance of deafness in affected dogs. Most anxious dogs gradually became bilaterally deaf by 3 months of age, later than is seen with pigment-associated CS deafness, but not all were affected, suggesting incomplete penetrance. The cochlear degeneration pattern is of the neuroepithelial type (27). Pedigree analysis suggested AR inheritance, but because of tight inbreeding, other mechanisms of inheritance could not be ruled out (143). Deafness does not seem to develop in pointers unrelated to this specific kindred.

Henthorn et al. (145) performed whole-genome scans on 59 pointers from a deaf pointer pedigree, half of which were affected. Significant linkage with deafness was located for three markers with LOD scores greater than 6 on CFA4. The syntenic canine gene for *cadherin 23* (*CDH23*), which causes the human non-syndromic recessive deafness DFNB12 and USH1D, was predicted to reside on the identified CFA4 segment. Sequencing the 69 exons of the *CDH23* gene from one affected dog identified one silent and one missense substitution in the gene’s open reading frame; the missense mutation was a substitution of proline to serine. There was 100% correlation between hearing or deafness and the substitution in over 80 dogs in the pedigree. This appears to be the first confirmed deafness gene identified in dogs.

##### Border collie

Border collies carry recessive alleles of the *S* locus and are subject to congenital hereditary CS deafness (76, 77). As a consequence, it took some time to recognize that a late onset form of deafness is also present in the breed. From a screening study of 216 adult dogs (146), five dogs out of three families that were over 12 years of age were identified with hearing loss using BAER testing; one was bilaterally deaf and four had reduced hearing. The loss had begun at about 5 years of age, which ruled out presbycusis as a likely cause, and several had been BAER tested as hearing at earlier ages. The loss was gradual, had no gender predilection, and appeared to follow an AD pattern (146, 147).

A genome-wide association study of 20 affected and 28 control dogs identified a significantly associated locus on chromosome 6 (CFA6) (147). Sequencing of affected and unaffected dogs identified two genes. *Ubiquitin specific peptidase 31* (*USP31*), which regulates NF-ĸB activation by members of the TNF receptor superfamily (148) was strongly associated with adult-onset deafness, suggesting involvement of the NF-ĸB pathway. NF-ĸB deficiency has been linked to hearing loss (149). *Retinoblastoma-binding protein 6* (*RBBP6*, OMIM entry 600938) was also implicated. A *RBBP6* knockout mouse model has been shown to have impaired hearing and the gene plays a probable role in ear development (150). The novelty of these genes as potential causes of late onset deafness in animals suggests that replication of the work would be beneficial.

##### Rhodesian ridgeback

A pattern has been reported in Rhodesian ridgebacks of a late onset of progressive deafness that may begin at 4 months of age and that reaches complete bilateral deafness by 12–18 months; males may exhibit onset earlier than females (151). Affected dogs have been identified in North America, Europe, Africa, and the United Kingdom. A non-peer reviewed source^3^ has reported that a genetic cause for the disorder, which is said to be AR, has been identified, but the gene identity and location have not been reported. Because of the lack of white pigment genes in the breed it is assumed to be NE pathology, but no anatomic studies have been reported. The available DNA test for Labrador retriever deafness does not detect deaf Rhodesians, so a different mutation on the same or a different gene must be responsible.

##### Cavalier King Charles spaniels

A form of late onset conductive hearing loss known as primary secretory otitis media (PSOM) appears in Cavalier King Charles spaniels, although at least one case each has also been reported in a Dachshund, a boxer, and a Shih Tzû (152). No proven hereditary cause has been identified, but the preponderance of occurrence in the one breed supports this as a possible factor. Several single-gene otitis media-causing mouse mutants have been identified and there is a suggestion of a hereditary component in OM in humans (153). A correlation between PSOM and pharyngeal conformation has been suggested (154), possibly from a thick soft palate and reduced nasopharyngeal aperture, which might argue against a hereditary basis. Because the phenotype of the Cavalier includes white pigment that probably results from a recessive allele of the *S* locus, affected dogs may incorrectly be thought to exhibit the congenital CS pathology.

Primary secretory otitis media, also known as glue ear, results from the development of a solid viscous plug in the middle ear due to either increased production of mucus by the middle ear lining or reduced drainage through the auditory (Eustachian) tube (152, 155, 156). Signs include hearing loss, neck scratching, otic pruritus, head shaking, abnormal yawning, head tilt, facial paralysis, or vestibular disturbances (156). Testing has confirmed that the hearing loss is conductive (157). Implantation of tympanostomy tubes has been successful in a small number of cases (158), but treatment is typically by myringotomy followed by flushing of the mucus plug, repeated as needed. No studies have been reported of genetic association studies.

### Cats

Along with deafness in Dalmatians, deafness in blue-eyed white (BEW) cats is perhaps the most recognized form of deafness in animals. Most hereditary deafness in cats is of the CS pathology, although some NE pathology associated with vestibular signs may also exist.

#### Dominant White Locus (*W*), White Spotting Locus (*S*)

Because of the prevalence of deafness in white cats, numerous studies have discussed or examined the condition, including Darwin in his seminal work *The Origin of Species* (29, 30, 159–174). Although the cochlear pathology has been shown to be CS in numerous studies (29, 30, 165, 168–171), a recent study has indicated that more than one type of cochlear pathology may exist (172, 173).

The Mendelian locus assignments for white color in cats are unsettled. The traditional view posited two loci: *dominant white* (*W*) and *white spotting* (*S*) (175, 176). *W* is autosomal-dominant over color and is unrelated to albinism. Cats with *W* are all or mostly white, with some exhibiting a colored spot on the head that may fade or disappear with age. White animals can be *WW* or *Ww*, but the homozygous cats do not appear to develop other disorders in the way that homozygous merle dogs can. Deafness can be unilateral or bilateral, and one or two blue eyes are frequently present, with the likelihood of deafness increasing with increasing number of blue eyes (175). Non-*W* cats can have blue eyes from the *c^s^* Siamese dilution pigment locus (Table 4), but deafness does not appear to be associated with this locus. Most early deafness studies were of cats with the *dominant white* locus.

The white spotting locus (*S*) presents with either recessive full solid color (*ss*), bicolor or ventral white which is dominant to solid color (*Ss*), or the dominant van color pattern (*SS*), named for cats from the Lake Van region in Turkey, where the only color is on the head and tail (177). Blue eyes are not typically seen and deafness does not appear to be associated with *S*.

*Dominant white* (*W*) may be allelic to *white spotting* (*S*), but epistasis has complicated a consensus terminology. Recently, the two have been combined into a single *white spotting* locus (*W*) that has pleiotropic effects of complete penetrance for a solid white coat and incomplete penetrance for deafness and blue iris color (60) (Table 4). The locus has four alleles: three dominant alleles – solid white (*W*) > high degree of spotting white (*W^h^*) > low degree of spotting white (*W^l^*) – and a single wild-type recessive allele of solid color (*w*), with *W^h^* and *W^l^* equivalent to *SS* and *Ss*, respectively. This terminology structure is supported by recent genomic studies (below).

Three studies of non-pure-breed white cats (*N* = 256) [(29, 165, 167); review by (174)] found a deafness prevalence of 12.1% unilateral and 37.9% bilateral, or 50% affected. In white cats out of two white parents, the combined prevalence was 52–96% across studies. Combined deafness prevalence was 85% or 65% in cats with two blue eyes, 40% in cats with one blue eye, and 22% or 17% in cats with no blue eyes. The deafness in white cats can be partial or complete for a given ear (169). Little prevalence data are available for pure-breed cats, but limited numbers have been provided for three breeds (178), including white and colored cats, based on behavioral tests. Prevalence was 18% in Norwegian forest cats (*N* = 329), 17% in Maine coon cats (*N* = 134), and 11% in Turkish Angora cats (*N* = 474). Iris color and coat color distributions were not reported, and percentages are likely underestimates because behavioral testing would not have identified unilaterally deaf cats. Another study of deafness prevalence in 84 white cats from 10 pure breeds (179) found similar prevalence rates of 9.5% unilaterally deaf and 10.7% bilaterally deaf, or 20.2% affected, based on BAER testing. Deaf cats were seen in 6 of the 10 breeds: Turkish Angora, British shorthair, Maine coon, Norwegian forest, Persian, and foreign white. No sex differences were seen, but blue-eyed cats were more likely to be deaf than non-blue-eyed cats.

A study of a cat colony with mostly white cats (*N* = 104) attempted to determine deafness inheritance using complex segregation analysis (178). The authors concluded that deafness was inherited as a pleiotropic gene segregating for deafness and blue irises with other polygenic effects.

A microsatellite marker study (180) identified a locus for *S* near the *KIT* gene on feline chromosome B1 and ruled out *EDNRB* from playing a role in white coloration. Subsequent studies (181, 182) demonstrated that both *dominant white* and *white spotting* resulted from insertions of *feline endogenous retrovirus 1* (*FEV1*) in intron 1 of *KIT*. Endogenous retroviruses are copies of exogenous retroviral genomes inserted into the host genome from ancestral infections and integrations into the genome (182). The white spotting pattern resulted from insertion of the full-length 7,125-bp *FEV1*, while the dominant white pattern resulted from insertion of a 623-bp fragment of *FEV1* at the same position. The retrotransposon insertion responsible for the merle locus in dogs (104, 113) is another example of a retroviral insertion affecting pigmentation. A specific gene mutation responsible for deafness in white cats has nevertheless not yet been confirmed.

#### Neuroepithelial Deafness

Very little is known of NE pathology deafness in cats, but cases of combined deafness and vestibular disease have been reported in Siamese and Burmese kittens (133, 136). Vestibular signs appear at or shortly after birth, are non-progressive, and may disappear with time; some cats were deaf but testing was not routinely applied.

### Other species

Deafness in dogs and cats has garnered more attention than similar problems in other species, largely due to the greater role played by pets in the lives of most people. Nevertheless, deafness can affect other species that are shown in competition or that otherwise have an economic importance, where deafness may have a deleterious impact. In some of the cases presented below, deafness has not been identified as being present due to difficulties in identification, but the genes discussed produce white patterns similar to those associated with CS pathology in dogs and cats, and mutations in those genes have produced deafness in humans, rodents, or other species.

#### Equids

Deafness has long been recognized to occur in horses with great amounts of white pigment and blue eyes (183), especially among Appaloosa, American Paint, piebald, skewbald, Pinto, and Clydesdale breeds and color patterns, suggesting similarity to pigmentation-associated patterns in dogs and cats. Depending on breed, numerous designations exist for white spotting patterns in horses, including chestnut, frame overo, cream, black, silver dapple, sabino-1 spotting, tobiano spotting, and dominant white (184). Seventeen white coat color genes have been identified and characterized in horses (185).

The lethal white foal syndrome (or overo lethal white syndrome, OLWS), which includes congenital aganglionosis, results from a defect in the *EDNRB* gene (118), and has been likened to a form of Hirschsprung disease in humans (186) that is associated with Waardenburg syndrome type 4A (WS4A) and that also results from an *EDNRB* mutation. Breedings of horses with white spotting patterns occasionally produce all-white or nearly all-white foals that typically die shortly after birth from the aganglionosis. Many have blue eyes and a few that have been hearing tested were deaf (187). Seven affected foals were shown to be homozygous for a 2-bp missense mutation in the first transmembrane domain of *EDNRB*, resulting in a lysine-for-isoleucine substitution at residue 118 of the gene (Ile118Lys); all tested frame overo horses (*N* = 40) were heterozygous for the mutation (118). Some horses without the overo pattern were heterozygotes, indicating variable penetrance of the mutation. Another study genotyped 945 white-patterned horses for residue 118 genotype and identified homozygous Ile118, homozygous Lys118, and heterozygous animals; all OLWS foals were homozygous for the Ile118Lys mutation but no homozygous adults were seen, reinforcing the suggestion of lethality of the homozygous pattern (188). Horses with the white patterns with the greatest amount of white (frame overo, highly white calico overo, and frame blend overo) had the highest incidence of the mutation, while horses with white patterns with low amounts of white (tobiano, sabino, minimally white calico overo, and breeding-stock solid) had the lowest incidence of the mutation. It is not clear that the Ile118Lys mutation is responsible for deafness.

A case report of a deaf overo American Paint horse (189) led to assessment of hearing status, eye color, and *EDNRB* genotype in 47 American Paint horses and pintos that were either deaf, suspected-deaf, or non-deaf (119). All deaf horses had one or usually two blue eyes and were heavily white marked. Thirty-one of 34 deaf and suspected-deaf horses were heterozygous for the *EDNRB* mutation, suggesting a strong association between the *EDNRB* mutation and what was probably congenital deafness.

A Franches-Montagnes colt was reported with heavy white markings and blue eyes that was born from parents with the bay coat color phenotype (190). BAER testing at 2 years of age demonstrated bilateral deafness. A *de novo* mutation in the *MITF* gene was shown to be responsible. Due to castration of the horse, the mutation has not been propagated.

Several studies have reported white pigmentation gene mutations, both with and without disease association, in horses and donkeys. Auditory studies have not been reported, so associations with deafness have not been excluded. Congenital stationary night blindness (CSNB) in Appaloosa horses, associated with a pigmentation pattern known as the leopard complex (*LP*) that is thought to be incomplete autosomal-dominant inheritance (191). The defect has been shown to result from significantly reduced expression of the *TRPM1* gene (*transient receptor potential cation channel, subfamily M, member 1*) in the retina and skin (184). However, deafness does not appear to be associated with CSNB, and *TRPM1* mutations have been associated with visual system disorders but not auditory disorders in other species (192). White pigmentation in horses but without reported hearing loss has also been shown to result from mutations in *MITF* and *PAX3* (193) and in *KIT* (194–196).

In other equids, two different mutations in *KIT* have been shown to be responsible for the dominant white pattern, a rare color pattern, and a different white spotting pattern in the donkey (197). A missense variant in exon 4 of *KIT* was seen in solid white donkeys, while a variant affecting a splice donor site was identified in donkeys with white spotting. Eighty-eight differences were identified in all 21 KIT exons between donkey and horse reference DNA. The authors did not report if any hearing disorders were present in the tested animals. Variants in *KIT* are responsible for sabino-1, tobiano, and dominant white patterns in horses (185).

#### Bovids

Early reports of deafness in cattle resulted from efforts by a Kansas Hereford owner to breed white beef cattle from a proband heifer purchased in dam in 1951 and described as an albino (198), although it was not clear that the animal was a true albino. The heifer was bred to a Hereford bull, producing a white, glass-eyed (blue iris) bull calf. This bull was in turn bred and produced normal calves and white, glass-eyed calves. One white bull was kept and bred to Herefords, Holsteins, and Guernseys, as well as descendants of original affected Herefords, producing only white, glass-eyed progeny. At the time of the report, the owner’s herd of 90 included 60 glass-eyed “albinos”. The owner described the whites as exhibiting photophobia and some deafness, but only behavioral assessment methods were available at that time. Most were pure white without spots or lightly pigmented areas. The skin, muzzle, eyelids, vulva, anus, and udder were pink, and the hooves were clear yellow, although two calves had brown spots on their shoulders. The irises were blue instead of the pink associated with most forms of albinism, and three calves had heterochromia irides (HI). The trait was suggested to be AD based on the owner’s recollections of breedings, which could suggest a *de novo* gene defect in the proband.

An unrelated Hereford herd, also in Kansas, had two albinos, a cow and her son, both with irregularly pigmented and non-­concordant irises (199). Eighty-one animals from the herd displayed three phenotypes of albinos, animals with normal coat colors but with HI, and normally pigmented animals. The HI animals had concentric ringed bicolored irises, with blue on the central ring and brown on the outer; half had the pattern bilaterally and half were unalike. No testing of auditory function was performed, so association with deafness is unknown. The authors argued for a dominant inheritance based on pedigree analysis, but could not entirely rule out recessive inheritance (199, 200). HI has also been reported in water buffalos (201), but no auditory testing was reported.

A recent study reported a white coat phenotype in German Fleckvieh cattle (202). Affected animals exhibited pure white coat color, pink skin, yellow hooves, white horns, absence of pigment on the muzzle, anus, eyelids, eye lashes, cilia, nictitating membranes, and conjunctiva, had blue irides, and were bilaterally deaf on behavioral and BAER testing. The pattern was inherited as AD with full penetrance and could be traced to a single proband dam. A genome-wide association study using a high-density SNP microarray identified a locus on bovine chromosome 22 (BTA22) that was shown by sequencing to be *MITF*; chromosome location had permitted ruling out *KIT, KITLG, PMEL, TYR, PAX3, EDNRB, EDN3*, and *SOX10* based on identified locations on the bovine genome on BTA2, 5, 6, 12, 15, and 29 (202). The mutation was a missense mutation in exon 7 of the bovine *MITF* M-form, for which all German White Fleckvieh animals were heterozygous and which was absent from 383 partially white controls from 10 different breeds.

A mutation in *KIT* has been shown to be responsible for a white pattern in domestic yaks (203), but hearing loss was not reported as being associated with the mutation.

#### Pigs

A CS pathology deafness pattern has been reported in the white Chinese Rongchang pig breed (204). Animals were profoundly deaf, demonstrated progressive loss of outer and inner hair cells, and had a deficiency of melanocytes in the stria vascularis. Seven mutations were identified in the M-promoter of *MITF* which downregulate gene transcripts. An earlier study (205) had ruled out *KIT* as the gene responsible for white in the breed.

#### Sheep

Hereditary deafness has not been reported in sheep, but a white/hypopigmentation color pattern has been shown to result from a homozygous deletion of the complete *EDNRB* gene (206).

#### Ferrets

Ferrets present in a variety of color patterns. Three first-level color patterns are colored (non-white), colored with white markings, and albino. Colored ferrets are split into sable, pastel, black, and chocolate, and coats with white markings are split into panda, American panda, blaze, silver, dark-eyed white (DEW), and mitt, all with white feet (207). A variety of additional coloration descriptors are also used by breed fanciers. From BAER testing of 152 pet ferrets with a variety of color marking patterns, 10 (7%) were unilaterally deaf and 34 (22%) were bilaterally deaf, or 29% affected regardless of color. One of 10 (10%) albinos was bilaterally deaf, which may have reflected the presence of an underlying white spotting pattern obscured by the epistatic effect of albino over other colors. Of all animals with any white markings (*N* = 79), 10 (13%) were unilaterally deaf and 33 (41%) were bilaterally deaf, or 54% affected. All panda (*N* = 11), American panda (*N* = 7), and blaze (*N* = 9) ferrets were deaf. None of the colored animals without white markings (*N* = 63) had any deafness (207). In animals with a pigmentation trait called premature graying (*N* = 23, out of 106 ferrets for which longitudinal data were available), 2 (9%) were unilaterally deaf and 18 (78%) were bilaterally deaf, or 87% affected. No gender differences were seen.

The albino pattern in ferrets has been shown to result from a deletion of exon 4 of the *TYR* gene (208), but the genetics of the inheritance of other coat colors in the ferret have not been characterized (209), so it is unclear what gene might be affected in the white marked colored animals and act on auditory function.

#### Mink

Mink have long been commercially raised on ranches for the production of fur for use in clothing; mink oil made from subcutaneous fat is also used in cosmetics and medicine. Selective breeding has produced a variety of coat colors, including dark brown, brown, beige, gray, and white. White mink display early onset deafness that appears within 7 days after the appearance of hearing, which is typically at 31 days (210). The structure and histopathology of the cochlea, which demonstrates CS pathology, has been well-studied (36, 37, 211). The Hedlund phenotype of the white American mink includes white coat, dark or blue irises, and deafness, and is said to be controlled by a simple incompletely recessive locus that is associated with deafness in the homozygous status (*hh*), and hearing and white markings on the belly, tail, and paws and dark color elsewhere in heterozygotes (*Hh*) (212). Targeting the mink *MITF* gene, two microsatellite markers were identified within the predicted gene M-promoter region; genotyping of 25 members from a half-sib family showed 100% cosegregation with the deaf white phenotype, providing strong evidence for *MITF* as causative for the deafness and white phenotype (212). However, no mutation in the gene sequence could be identified. Histologic studies of the cochleas from normal and affected animals demonstrated the presence of melanin granules in the stria of normals but a complete absence of granules in deaf whites, supporting a CS mechanism for the deafness that had been established in earlier studies.

#### Camelids

Hearing tests using the BAER in llamas in Germany and alpacas in Australia (*N* = 63 total) identified a correlation between white coat, blue eyes, and deafness (213). Thirteen animals had bilateral blue eyes and one had HI. No animals with pigmented eyes and coats were deaf. Seven of 10 BEW animals were bilaterally deaf and one was unilaterally deaf. Two BEW animals had normal hearing, and three blue-eyed animals with pigmented coats had normal hearing. All white animals with normal eye pigmentation and one with HI had normal hearing.

Genomic study identified a strong association between the BEW phenotype in alpacas and the *KIT* gene (214). Two *KIT* alleles were suggested to be necessary: homozygosity of an allele named *bew2* was required for the white coat, and the additional presence of the allele *bew1* was required for the BEW phenotype. The presence of the *bew1* allele alone produced gray animals.

#### Rabbits

Deafness occurs in pigmented and white pet rabbits, but the condition has not been studied or well characterized. Hereditary deafness has not been reported, but the white pigmentation color pattern has been shown to result from the *KIT* gene. The *Dominant white spotting* locus or *English spotting* locus (*En*) is an incompletely dominant trait that produces complete white when homozygous (*EnEn*) and a normally spotted pattern in heterozygous (*enEn*) animals. Homozygous white animals have a low viability because of the presence of a grossly dilated cecum and ascending colon, similar to the aganglionosis of lethal white foal syndrome of horses (118) and the human Hirschsprung disease (186) that is associated with a form of Waardenburg syndrome. A single nucleotide polymorphism in *KIT* completely cosegregated with the phenotype (215), and *KIT* expression in the colon and cecum of affected rabbits was 5–10% that of pigmented rabbits. An earlier study (216) had ruled out *EDNRB* as a cause. Cochlear tissues were not examined and there is no reported indication that affected rabbits have any hearing disorder despite the similarities to lethal white foal syndrome and Hirschsprung disease. HI has been reported in a pigmented Dutch rabbit (217), but deafness was not present.

## Summary

Although the piebald locus is caused by *MITF* and the merle locus is caused by *PMEL* in dogs, and the white spotting locus is caused by *KIT* in cats, the genes responsible for the pigment-associated deafness in these species have not yet been identified. The gene responsible for deafness in nervous pointers was identified as *CDH23* (144), but these dogs are from a limited research pool. *USP31* and *RBBP6* have been shown to be strongly associated with late onset deafness in border collies. *EDNRB* is probably responsible for deafness in lethal white foal syndrome (117, 187) and deafness in BEW American Paint and pinto horses (118). Mutations in *MITF* were shown to be responsible for deafness in a Franches-Montagnes colt (189) and in German Fleckvieh cattle (201), Chinese Rongchang pigs (203), and Hedlund white American mink (210). Despite this progress, much work remains in identifying unequivocal gene mutations that can be shown to be directly causative of deafness. In animals that are bred and raised for show and where a mandated phenotype is strongly associated with deafness, such as a white coat and absence of a patch in the Dalmatian, progress in reducing deafness prevalence will by necessity be slow, but will be facilitated by the availability of a DNA marker that can guide breeding decisions.

## Conflict of Interest Statement

The author declares that this work was conducted in the absence of any commercial or financial relationships that could be construed as a potential conflict of interest.

## Footnotes

^1^http://www.thekennelclub.org.uk/media/16446/flatcoated%20retriever.pdf

^2^https://projectdog.org/order/dings. Accessed 5/29/15.

^3^“The hereditary basis of early onset adult deafness in the Rhodesian ridgeback breed.” https://projectdog.org/projects/ridgeback_deafness. Accessed 5/29/15.
